# Birds in space and time: genetic changes accompanying anthropogenic habitat fragmentation in the endangered black-capped vireo (*Vireo atricapilla*)

**DOI:** 10.1111/j.1752-4571.2011.00233.x

**Published:** 2012-01-24

**Authors:** Giridhar Athrey, Kelly R Barr, Richard F Lance, Paul L Leberg

**Affiliations:** 1 Department of Biology, University of Louisiana at LafayetteLafayette, LA, USA; 2 Environmental Laboratory, US Army Engineer Research and Development CenterVicksburg, MS, USA

**Keywords:** anthropogenic fragmentation, bottlenecks, evolutionary dynamics, fragmentation, genetic differentiation, genetic diversity

## Abstract

Anthropogenic alterations in the natural environment can be a potent evolutionary force. For species that have specific habitat requirements, habitat loss can result in substantial genetic effects, potentially impeding future adaptability and evolution. The endangered black-capped vireo (*Vireo atricapilla*) suffered a substantial contraction of breeding habitat and population size during much of the 20th century. In a previous study, we reported significant differentiation between remnant populations, but failed to recover a strong genetic signal of bottlenecks. In this study, we used a combination of historical and contemporary sampling from Oklahoma and Texas to (i) determine whether population structure and genetic diversity have changed over time and (ii) evaluate alternate demographic hypotheses using approximate Bayesian computation (ABC). We found lower genetic diversity and increased differentiation in contemporary samples compared to historical samples, indicating nontrivial impacts of fragmentation. ABC analysis suggests a bottleneck having occurred in the early part of the 20th century, resulting in a magnitude decline in effective population size. Genetic monitoring with temporally spaced samples, such as used in this study, can be highly informative for assessing the genetic impacts of anthropogenic fragmentation on threatened or endangered species, as well as revealing the dynamics of small populations over time.

## Introduction

Among the challenges to understanding evolution at the population level is unveiling demographic history using genetic data. Although ‘snapshots’ of genetic patterns based on single samples can be extremely informative, the ability to divulge historical population processes that shaped current patterns of genetic variation is limited (Beaumont 2003; Miller and Waits 2003). This can be complicated by the fact that alternative evolutionary histories potentially result in similar genetic estimates. Accurately characterizing evolutionary or demographic history is especially valuable, as it can inform us on how the past can influence the future trajectory of populations: for example, if populations have been isolated or persisting at small sizes, they could accumulate inbreeding (Frankham 1995a, b) or lose adaptive genetic potential (Willi et al. 2006), interfering with future evolution (Ellstrand and Elam 1993; Holsinger 2000). Populations lacking or with reduced gene flow could eventually fix different alleles (drift) – adaptively diverging, or may instead suffer local extinction (Maruyama and Kimura 1980; Hanski 1998; Reinhardt et al. 2005). Low genetic variation resulting from historically small population sizes, in contrast to that resulting from a recent bottleneck, may have different outcomes for the species (Spencer et al. 2000; England et al. 2003). Knowledge of what ancestral conditions resulted in current states helps us better understand the evolutionary implications of demographic events – even as they may be unfolding.

This need to understand the potential evolutionary outcomes of past demography is nowhere as crucial, perhaps, as in cases where the genetic impacts of anthropogenic actions need to be assessed – as is often the need in conservation studies. For species existing in small populations and those of conservation concern, it may be necessary to unravel demographic history to evaluate whether genetic sustainability is of concern (Beaumont 1999; Gaggiotti et al. 2004), and to determine the extent to which habitat change can influence genetic change. Unfortunately, reconstructing historical genetic patterns and processes based on contemporary genetic patterns can be challenging, primarily because of the lack of reference samples spanning temporal and spatial ranges for species of interest.

One avenue for circumventing the above limitations is to use archived specimens and molecular techniques sufficiently sensitive for working with limited or degraded DNA (Ellegren 1991; Mundy et al. 1997a; Nielsen and Hansen 2008). Such samples make it possible to assess whether population genetic diversity has declined and genetic structure has increased and to explore demographic scenarios that best explain observable genetic patterns (Johnson et al. 2004; Martinez-Cruz et al. 2007). Knowledge of demographic history obtained by this approach can, for example, by helping guide our efforts in managing populations of endangered species, by delivering rapid, conservation relevant information. Here, we report on a study investigating genetic and demographic change over 100 years in populations of black-capped vireos (*Vireo atricapilla*) – an endangered bird species.

The black-capped vireo is a neotropical songbird that breeds in the early-successional oak-juniper savanna ranging from south-central Texas through southern Oklahoma (USA). This species is reported to have suffered substantial losses in both its breeding and wintering habitat because of agriculture and urbanization over the past 50 years (USFWS 1991). At the time of listing as an endangered species, the estimated breeding population in the United States was about 190 pairs. Although historical census estimates are not available, it was thought at the time of listing that the population size had undergone a substantial contraction (USFWS 1991). Historical habitat assessments are unavailable, but just between 1992 and 2002, up to 8.6% of suitable rangeland (3.2 million acres) was converted to pastures or croplands. During the same period, ranch ownership increased by approximately 20 000 new farms (of 500 acres or less), changing the contiguity of rangeland and fragmenting both land ownership and habitat areas (Wilkins et al. 2006). In addition to anthropogenic habitat fragmentation, suppression of natural fires and high rates of brood parasitism by the brown-headed cowbird (*Molothrus ater*), associated with increasing habitat edges, are considered as important influences. Severe parasitism by cowbirds is considered to be a relatively recent problem – having increased together with fragmentation – although it is reasonable to expect that some parasitism has always existed.

Recently, we reported that many populations of black-capped vireos showed more structure than might be expected, based on the vagility of these birds (Barr et al. 2008). There was little evidence for loss of genetic diversity within populations owing to strong bottlenecks, considering the population estimates in 1991. We speculated that the observed genetic structure was a recent occurrence caused by fragmentation and that genetic differentiation might be more sensitive to habitat fragmentation and population reductions than measures of genetic diversity (Barr et al. 2008; Leberg et al. 2009). Contemporary sampling alone, however, was not enough to eliminate the possibility that the observed pattern is the historical norm. Current populations appear stable or in some cases increasing as a result of localized habitat management (prescribed burns, limited grazing) and cowbird control (USFWS 1991; Wilkins et al. 2006). Habitat loss and fragmentation continue, however, and semi-isolated remnant populations are susceptible to stochastic processes (Charlesworth et al. 2003; Wang 2005). It may be beneficial to determine whether the genetic consequences of past or ongoing isolation effects may become a concern for future viability of remnant populations, despite management efforts.

Little is known about the demographic history of this species, and especially about past genetic diversity and population sizes. In this study, we (i) compare diversity indices and population structure from historical and contemporary populations, (ii) use approximate Bayesian computation models to explore the demographic history – especially with regard to the amplitude and timing of putative population size changes and (iii) estimate long-term effective population size (*N*_ev_) to understand the impact of drift on remnant populations.

## Materials and methods

### Locations and times of samples for temporal analyses

**Figure 1:**
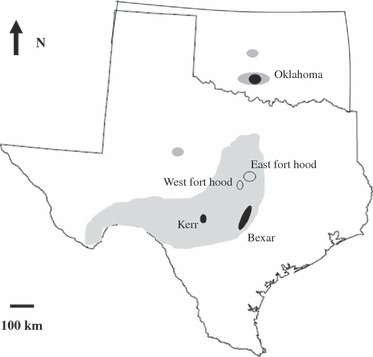
A map showing the distribution of breeding habitats of the black-capped vireo in central Texas and south-central Oklahoma. The shaded regions denote the historical breeding range of the species, but the current breeding range is considerably limited (not depicted). Filled ovals represent sites where we have both historical and contemporary samples.

We focused on the availability of multiple samples collected from 1899 to 1915 at locations from areas corresponding with the contemporary samples collected during 2005–2008. We chose this period because (i) few samples existed in collections either before or after this time from multiple locations and (ii) the sampling straddles the most likely period of habitat and population decline. Three locations (Fig. 1) represented by museum-archived material meet these criteria: Kerr County, TX, Travis-Comal-Bexar counties, TX, and Blaine-Caddo-Comanche counties, OK (hereafter referred to as Kerr, Bexar and Oklahoma, respectively, and generally as historical populations). Currently, censuses for these sites are approximately 500, 130 and 2000 territorial pairs, respectively (Barr et al. 2008). Analyses of these three sets of populations will hereafter be referred to as historical–contemporary comparisons (Table 1). For all analyses, we used microsatellite markers developed specifically for this species (Barr et al. 2007).

**Table 1:** Classification of comparisons and the time points over which we assessed changes in genetic diversity. *N*_e_ estimation was based on the historical–contemporary samples

Comparison	Site	Year
Historical	Kerr	1900, 1910, 1915
Historical–contemporary	Bexar*	1910‡, 2005
	Kerr	1900, 1910, 1915, 2005
	Oklahoma†	1910‡, 2005
Contemporary	Kerr	2005, 2008

* Bexar was the combined sample from Bexar–Travis–Williamson counties (sites SA and BC in Barr et al. 2008).

† Oklahoma was the combined sample from Blaine–Caddo–Comanche counties.

‡ Samples labeled 1910 for Bexar and Oklahoma consist of those collected from 1906 to 1910.

### Contemporary sample processing

Contemporary samples were collected in 2004–2006, with additional samples collected in 2007 and 2008. Methodology of sample collection is similar to the one described by Barr et al. (2008). In brief, sampled individuals were territorial adults, and occasionally juveniles. Individuals were captured using conspecific playback, and all individuals were captured during the breeding season for this species between April and July. Contemporary samples were genotyped with the same methodology as that for the historical samples, except where noted.

### Historical sample collection and genotyping

Historical sample DNA was extracted from 1-mm^3^ patch of skin removed from the toe-pads of museum specimens using sterile surgical razors and then placed in a sterile microcentrifuge tube (Mundy et al. 1997a, b). We adhered to the recommendations of Bonin (Bonin et al. 2004; Pompanon et al. 2005) to prevent contamination, reduce and estimate genotyping error rates, and establish the reliability of genotyping protocols. Details of laboratory techniques are presented in Appendix S1.

Prior to quantification of reliability and estimation of error rates, we tentatively assigned reliability if 3 out of 4 replicate PCRs produced the same results.

### Establishing reliability and estimating error rates

We used Microchecker (Van Oosterhout et al. 2004) to proofread our data for entry errors and null alleles. To establish the reliability of our genotyping, we used the program RELIOTYPE (Miller et al. 2002). This program uses a maximum likelihood approach for reducing genotyping errors and provides recommendations for replications at each locus to reach a ≥95% reliability level. Once individual loci–sample combinations exceeded this reliability level, the data can be considered acceptable without further replication. We also used the program GIMLET (Valière 2002) to estimate the errors arising as a result of allelic dropout across all replicates and to obtain a ‘consensus’ data set on which statistical analysis could be conducted.

### Statistical analysis

We estimated allele frequencies and performed exact tests for deviations from Hardy–Weinberg expectations using the online tool Genepop on the web (Raymond and Rousset 1995). For all samples, we estimated measures of unbiased expected heterozygosity (*H*_EXP_) using GENETIX (Belkhir et al. 2004). We used FSTAT (Goudet 2002) to estimate allelic richness (*A*_R_), which controls for biases in allelic diversity arising from unequal sample sizes (Leberg 2002).

To estimate changes in genetic diversity between a) historical and contemporary samples or b) between pairs of samples within the contemporary period, we used a Wilcoxon signed-rank test (PROC UNIVARIATE; Statistical Analysis Software, SAS Institute, Cary, NC, USA). This nonparametric analysis uses estimates of *H*_EXP_ and *A*_R_ by locus (loci are replicates) to test the hypothesis that the sample medians do not between differ time points. For historical comparisons, where we have three time points, we used a Kruskal–Wallis test (PROC NPAR1WAY; Statistical Analysis Software), blocked by locus, to compare *H*_EXP_ and *A*_R_ among sampling periods.

Historical population genetic structure was characterized by estimating pairwise *F*_ST_ (Θ of Weir and Cockerham) among the 1905–1910 historical samples from Kerr, Bexar and Oklahoma populations in GENETIX. *F*_ST_ estimates were obtained using 3000 permutation resampling to control for possible variation because of differences in sample sizes. We performed identical analyses on the corresponding three contemporary populations for comparison. We used a Wilcoxon signed-rank test (paired by locus) to see whether the degree of differentiation among historical samples was different from that of contemporary samples, for the three population pairs. Pairwise differentiation values for each locus were the replicates in the statistical comparison. Using *F*_ST_ to infer differentiation in cases where intra-population diversity is high can be problematic (Hedrick 1999); hence, we also estimated three other measures of population differentiation, namely *G*_ST_, 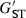 (Hedrick 2005) and *D*_EST_ (Jost 2008). We performed identical analyses on these estimates as on the *F*_ST_ estimates to determine whether levels of differentiation have increased between historical and contemporary periods.

### Approximate Bayesian computation of the timing and intensity of bottleneck

We used coalescent simulations of several alternate demographic histories to estimate the time at which putative bottleneck event(s) may have taken place and to estimate the historical and present population sizes. Coalescent inference methods can utilize genetic summary statistics to extract evolutionary histories (Beaumont et al. 2002; Ramakrishnan et al. 2005), and the posterior probabilities of specific scenarios can be estimated using approximate Bayesian computation (ABC). We felt that ABC was the best approach to investigate the demographic history and population size changes of our sample populations, because (i) utilizing summary statistics better accounts for demographic factors that impact genetics and (ii) existing *N*_e_ estimators are reported to not perform well for species with overlapping generations, small sample sizes or other assumptions, because of which pooling samples can be problematic (Luikart et al. 2010).

The ABC approach can be briefly summarized as follows: First, coalescent population gene genealogies are simulated from a uniform prior distribution for each parameter of interest. Priors are defined based on what is known from the species biology, or based on estimates for similar species. Then, genealogy and parameter space is explored through Markov chain Monte Carlo (MCMC) algorithms with a range of uniform priors, producing a large reference table of summary statistics from which genetic data are simulated. Second, a rejection algorithm using Beaumont et al. (2002) similarity criterion rejects all but the data sets closest to the observed data. Finally, posterior probabilities of scenarios and parameters are estimated. We implemented the coalescent simulation and parameter estimation using the program DIYABC (Cornuet et al. 2008). We combined all historical samples into one temporal sample and all contemporary samples into the second temporal sample, for two reasons: (i) we were interested in estimating the *N*_e_ and timing of the putative decline for the whole species and (ii) incorporating divergence events among sample sites in the coalescent genealogies would exclude gene flow between lineages, which we know to be incorrect.

We coded 11 potential scenarios to explore demographic history and to detect bottlenecks from our two temporal samples, and a total of 11 million data sets were simulated. A summary of scenarios evaluated and priors used are presented in Table 2. Following calculation of distances between observed and simulated data sets, we performed local logistic regression on 1% of the simulated data sets closest to the observed data to obtain posterior probabilities for tested scenarios, and estimate parameters (Beaumont et al. 2002; Cornuet et al. 2008).

**Table 2:** Description of the eleven scenarios that were evaluated using coalescent simulation and ABC estimation. In all but one case, the putative time at which population decline *t* generations ago was estimated

Scenario	Evaluated conditions	Notation	Parameters estimated
1	Constant or increasing size between ancestral (Na) at *t*_90_ and modern samples (N1) at *t*_0_	Na ≤ N1	Na:U[10:100 000] N1:U[10:100 000]
2	Population size declined from 4000 at *t*_90_ to 500 at *t*_0_	Nb > N2	Time of bottleneck t:U[10:80]
3	Population size declined from 5000 at *t*_90_ to 650 at *t*_0_	Nc > N3	Time of bottleneck t:U[10:80]
4	Population size declined from 6000 at *t*_90_ to 700 at *t*_0_	Nd > N4	Time of bottleneck t:U[10:80]
5	Populations size declined from 2000 at *t*_90_ to N5 at *t*_0_	Ne > N5	Time of bottleneck t:U[10:80] N5:U[200:1000]
6	Population size declined from 3000 at *t*_90_ to N6 at *t*_0_	Nf > N6	Time of bottleneck t:U[10:80] N6:U[200:1000]
7	Population size declined from 4000 at *t*_90_ to N7 at *t*_0_	Ng > N7	Time of bottleneck t:U[10:80] N7:U[200:1000]
8	Population size declined from 5000 at *t*_90_ to N8 at *t*_0_	Nh > N8	Time of bottleneck t:U[10:80] N8:U[200:1000]
9	Population size declined from Ni at *t*_90_ to N9 at *t*_0_	Ni > N9	Ni:U[2500:15 000] Time of bottleneck t:U[10:80] N9:U[100:1000]
10	Population size declined from Nj at *t*_90_ to N10 at *t*_0_	Nj > N10	Nj:U[2500:15 000] Time of bottleneck t:U[10:80] N10:U[100:1000]
11	Population size declined from Nk at *t*_90_ to N11 at *t*_0_	Nk > N11	Nk:U[2000:10 000] Time of bottleneck t:U[10:80] N11:U[500:750]

*t*_0_ represents our contemporary sample, and *t*_90_ refers to the historical sample. Scenario 1 is the null hypothesis of constant population or increasing size.

### Estimation of *N*_e_

We used temporal changes in allele frequencies to obtain information about *N*_e_ in our sampled populations. For microsatellite data, the temporal method is reported to be more precise when samples are taken several generations apart than point estimates (Waples and Do 2010). To concurrently estimate the impacts that drift and migration have had over our sampling interval, and considering our access to samples from multiple time points (Kerr), we chose to use the pseudo-maximum likelihood estimator (Wang 2001; Wang and Whitlock 2003) as implemented in the program MLNE. In this method, *N*_e_ is estimated jointly with the migration rate *m* (generally referred to as the joint estimate of ML*N*_*e*_), where it is assumed that migrants originate from an infinitely large population. This was important because, despite our finding of population differentiation, *F*_ST_ values indicate that the study populations experience gene flow (Barr et al. 2008). Additionally, we did not know, prior to this study, whether rates of gene flow have changed in response to demographic and habitat changes. Furthermore, there have been two observations of individuals moving far enough (70 km) to result in potential gene flow between our sampled populations (Kostecke and Cimprich 2008).

Concurrent estimation of *m* with *N*_e_ requires that a source population is specified from which gene flow may occur. We created source populations for each focal (analyzed) population by combining samples from the other two remaining populations. While the assumption (infinitely large source) is typical for many idealized populations, in reality few species satisfy this assumption. Although historical populations may not have been infinite, they may have represented a panmictic population, without significant population structure. Hence, we avoided using contemporary populations as the source population as gene flow from differentiated populations may produce biased estimates (Johnson et al. 2004). Temporal estimation of *N*_e_ based on the moment or likelihood approach assumes discrete generations. As black-capped vireos do not fit the assumption of discrete generations, and as we lack accurate information regarding generation lengths (*T*), we assumed three potentially different generation lengths (*T* = 1, 1.5 and 2 years) to evaluate the effects that *T* may have on estimates of *N*_e_. There is some variation among sexes (Graber 1961) regarding the ages at which they reproduce. Males only rarely maintain territories before their second year (breeding by nonterritorial, sub-adult males is suspected but not verified), whereas females breed in their first year.

For comparison with the joint estimation approach, we also obtained temporal estimates of *N*_e_ assuming closed populations using three approaches: (i) the moment estimator of *N*_e_ (*MtN*_*e*_) based on equations 15 and 18 from Nei and Tajima (1981), (ii) the pseudo-maximum likelihood estimator (Wang 2001) and (iii) the coalescent Monte Carlo estimator (Co*N*_*e*_) implemented in the program CoNe (Anderson 2005).

We also used the program ONeSAMP (Tallmon et al. 2008) to obtain a point estimate of *N*_e_ for the historical and for the contemporary populations, using the pooled samples as with the ABC approach. For ONeSAMP, we defined priors of [10–10 000] for both the historical and modern samples.

## Results

Reliability of genotyping protocols and error rates are reported in Appendix S1.

### Estimates of genetic diversity

All 9 loci used were polymorphic for both historical and contemporary samples. A total of 133 different alleles were detected in the historical samples, and 124 alleles were detected in the contemporary samples. There were no differences in allelic richness (*A*_R_) and heterozygosity (*H*_EXP_) among the 1905, 1910 and 1915 samples from Kerr (Table 3).

**Table 3:** Measures of mean multilocus genetic diversity (with SEs) for historical and present population of black-capped vireo from different sampling times. The hypothesis of no difference in estimates of expected heterozygosity (*H*_EXP_) or allelic richness (*A*_R_) was evaluated with a Wilcoxon signed-rank test or Kruskal–Wallis test*. Sample years with census size (*N*_e_) are given

Site	Sample year (*N*_c_)	*N*	*A*_R_	*Z* or H*	*P*	*H*_EXP_	Z or H*	*P*
Historical comparisons								
	1900	8	8.88 (0.512)	0.13	0.890	0.91 (0.012)	3.76	0.1526
Kerr	1910	15	8.56 (0.519)			0.87 (0.022)		
	1915	20	8.72 (0.388)			0.90 (0.008)		
Historical–contemporary comparisons								
Kerr	1915	20	8.72 (0.388)	2.429	0.008	0.90 (0.013)	2.54	0.005
	2005 (1000)	17	6.75 (0.496)			0.80 (0.034)		
Bexar	1906–1910	9	9.56 (0.765)	2.666	0.004	0.91 (0.011)	2.062	0.019
	2005 (260)	33	6.51 (0.619)			0.79 (0.031)		
Oklahoma	1906–1910	8	8.44 (0.647)	2.667	0.044	0.89 (0.021)	2.76	0.004
	2006 (4000)	34	6.00 (0.483)			0.75 (0.034)		
Contemporary comparisons								
Kerr	2005 (1000)	17	6.75 (0.496)	0.178	0.48	0.80 (0.021)	0.889	0.18
	2008 (1000)	25	6.29 (0.668)			0.81 (0.027)		

* For comparisons involving pairs of samples, the hypothesis of no difference between samples was evaluated using Wilcoxon signed-rank test; Kruskal–Wallis test was used in comparisons involving three temporally spaced samples.

Levels of *A*_R_ and *H*_EXP_ were significantly lower in contemporary samples than in the historical samples from the same sites (Table 3). Nine alleles were found in the historical samples (across all three populations) that were not found in any of the sampled modern populations, despite the larger number of the contemporary samples. Much larger changes in genetic diversity were observed between the historical and contemporary periods than between any of the samples collected prior to 1915 or since 2005.

### Population structure

Based on *F*_ST_, the historical Bexar and Kerr samples were not differentiated, whereas the Oklahoma sample was significantly differentiated from Kerr (Table 4). In contrast, all the corresponding contemporary populations were significantly differentiated. Results based on the other three estimators –*G*_ST_, 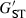 and *D*_EST_– also showed the same pattern of differentiation among historical and contemporary populations (Table S1). We also found that the degree of differentiation was significantly higher among the contemporary samples compared to the historical samples for all pairs of sites (Table 4), with population pairs showing on average a twofold increase in differentiation based on *F*_ST_. Similar comparisons using *G*_ST_, 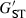 and *D*_EST_ also showed a significant increase in the degree of differentiation (Table S1), despite 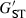 and *D*_EST_ values (adjusted for within-population diversity) being generally higher than *F*_ST_ and *G*_ST_ for both the historical and contemporary periods.

**Table 4:** Pairwise differentiation between the three pairs of sites that were sampled in both the historical and contemporary periods

	Historical samples		Contemporary samples		Historical v/s contemporary comparison	
			
Sample pair	*F*_ST_	P*	*F*_ST_	P*	*Z*	*P*†
Bexar–Kerr	0.0131	0.126	0.0461	0.003	2.71	0.004
Kerr–Oklahoma	0.0116	0.010	0.0348	0.003	2.87	0.003
Oklahoma–Bexar	0.0126	0.088	0.0497	0.003	2.66	0.004

Estimates of *F*_ST_, and *P* values for historical and contemporary comparisons are provided. To compare levels of historical versus contemporary differentiation, we used Wilcoxon signed-rank tests, and the respective *Z* and *P* values are shown.

* Test of hypothesis that *F*_ST_ is not different from zero.

† Test of hypothesis that *F*_ST_ from the historical period is not different than *F*_ST_ from the contemporary period.

### ABC estimation of parameters

**Figure 2:**
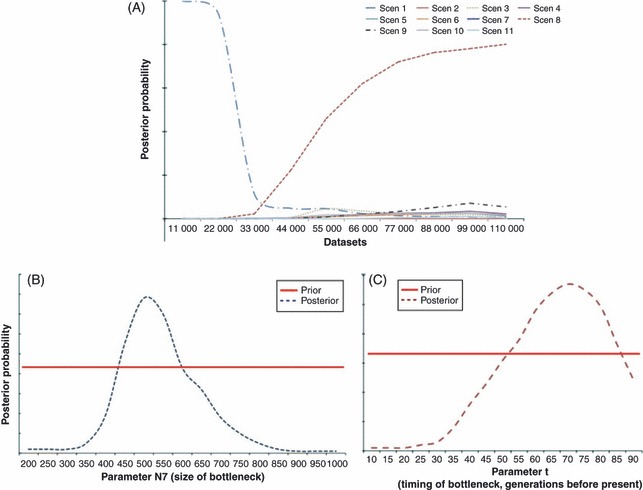
(A) Posterior probabilities of 11 evaluated scenarios, showing that scenario 8, requiring a decline in population size from 5000 to a lesser value (200–1000), was the best supported over a 110 000 closest data sets. (B) Posterior probability for the estimated parameter N8, which as estimated as 450 (95% CI, 320–821). (C) Posterior probability of an event *t* generations ago when bottleneck occurred (*x*-axis, generations 10–90). *t* was estimated to as 67 (95% CI, 36–81) generations before present.

There was no support for the scenario requiring a constant or increasing population size between the two sampled periods. Scenario 8 had the highest posterior probability among all scenarios tested, which required a population decline from 5000 at *t*_90_ to N8 [200:1000] at *t*_0_ (Fig. 2A). N8 was estimated to be 450 (0.025 and 0.975 credibility interval 310–821) (Fig. 2B), and the parameter *t*, the time at which a bottleneck occurred, was estimated to be 67 (0.025 and 0.975 credibility interval 36–81) generations ago (Fig. 2C).

### Estimates of effective population size (*N*_e_)

For all estimators, we have limited confidence in the *N*_e_ estimates we obtained for Oklahoma and Bexar populations owing to the small sample sizes on which these were based. Estimates obtained from small sample sizes cannot preclude the possibility of random sampling error and may not be representative of allele frequency changes over time.

Across our comparisons, likelihood estimates of *N*_e_ that jointly estimated *m* were much smaller than other estimators that assume closed populations (Table 5 and 6). The joint estimate of ML*N*_*e*_ also decreased as *T* increased. We will only report on results from tests assuming *T* = 1 and 2, as *T* = 1.5 produced intermediate values in all cases. The upper limit of the 95% CI was substantially affected by changes in *T*. Although the estimates of *N*_e_ are not independent of *T*, they are not quantitatively different as there was considerable overlap of 95% CIs for estimates based on *T* =1 and 2. To simplify our review and discussion of the results, we limit our discussion to estimates assuming *T* = 1, although the estimates for *T* = 2 are presented in supporting online material (Table S2).

**Table 5:** Temporal estimates of effective population size (*N*_e_) from historical–contemporary sampling and assuming generation lengths (*T*) of 1 year. Values shown are estimates of the likelihood estimates of *N*_e_ (ML*N*_*e*_), migration (*m*) and their 95% confidence intervals

Site	Time	No. Gen	ML*N*_*e*_	95% CI	*m*	95% CI
Historical–contemporary comparisons						
Bexar	1910–2005	95	38	17–208	0.081	0.012–0.243
Kerr	1915–2005	90	180	43–419	0.025	0.010–0.132
Oklahoma	1910–2005	95	31	15–180	0.074	0.017–0.206

**Table 6:** Temporal estimates of the effective population size (*N*_e_) based on historical and contemporary samples, assuming closed populations (no migration) and a generation length (*T*) of 1 year

Site	Time	No. Gen	Mt*N*e	ML*N*_*e*_	95% CI	*CoN*_*e*_	95% CI
Historical–contemporary comparisons							
Bexar	1910–2005	95	198	597	408–872	1263	803–2379
Kerr	1915–2005	90	242	552	394–809	792	554–1217
Oklahoma	1910–2005	95	129	418	308–5m84	722	513–1084

Values shown are estimates of the likelihood estimates of the moment estimate Mt*N*_*e*_, the likelihood estimate (ML*N*_e_), and the coalescent estimator Co*N*_*e*_. The corresponding 95% confidence intervals are shown.

The ML*N*_*e*_ estimates (jointly with *m*) for the Bexar and Oklahoma samples based on two sampling periods (1910–2005) were approximately 38 (95% CI, 17–208) and 31 (95% CI, 15–180), respectively (Table 5). For Kerr, the estimate for samples from 1915 to 2005 was 180 (95% CI, 43–419).

For estimates assuming closed populations, the three estimators were qualitatively similar with respect to the trends observed for each of the three populations. In summary, the moment estimator (Mt*N*_*e*_) produced the smallest estimates, coalescent estimator (Co*N*_*e*_) produced the largest estimates, and the likelihood estimator (ML*N*_*e*_, closed) produced estimates intermediate to the other two methods (Table 6).

Results from ONeSAMP gave *N*_e_ estimates of 9345 (95% CL, 7895–9870) for the historical population, and 872 (95% CL, 765–1265) for the modern population samples.

## Discussion

### Estimates of genetic variability

Measures of genetic variability in historical samples from Kerr did not vary between the years 1900 and 1915. Although it is impossible to say whether population sizes remained constant during this period (based on our samples), drastic demographic changes appear unlikely during 1900–1915. Allelic richness was higher in the historical samples compared to the contemporary samples, indicating a population contraction. Nine alleles were not detected in the contemporary samples compared to historical samples. No reciprocal condition of unique alleles in contemporary populations was detected. Allelic richness is considered more sensitive to population bottlenecks than is heterozygosity (Leberg 1992; Spencer et al. 2000). Estimates of *H*_EXP_ followed a similar pattern as *A*_R_, although the proportional change was smaller in the former. Our observation of lower heterozygosity in contemporary relative to the historical populations indicates that inbreeding or genetic drift had occurred (Frankham 1995a, b), probably due to a prolonged period of small population sizes spread over several generations (Nei et al. 1975) as it takes much longer for inbreeding to accumulate in populations compared to the time required to lose alleles by drift. These are important results, considering that our earlier work had failed to find evidence for bottlenecks based on current genetic samples alone (Barr et al. 2008). Although it is possible that similar declines in diversity could occur at constant population sizes, the probability of that is slim compared to a bottleneck explanation (see Appendix S2 for details of coalescent simulations to examine this).

### Population structure

This historical divergence between Texas and Oklahoma populations may represent a long established discontinuity in vireo habitat that pre-dated urbanization of the mid-20th century. Historical vireo distributions in Texas and Oklahoma are separated by approximately 400 km (Fig. 1) of unsuitable habitat, and this large gap might have been sufficient to result in historical genetic structure, although mtDNA results indicate a lack of phylogenetic structure (Zink et al. 2010). Our results indicate that the amount of differentiation among all sampled contemporary populations has significantly increased compared to historical populations. It appears that gene flow has declined between the historical and contemporary period – although it is difficult to say whether this change has been gradual or otherwise, based on only two temporal samples. Habitat loss and fragmentation – the candidate causes for demographic changes in black-capped vireo populations – have certainly been gradual over the past century, having accelerated after 1950. This finding is consistent with the analysis of DiBattista (2008), who reported that genetic variation typically declined in response to anthropogenic habitat fragmentation. An increasing number of recent studies, across various taxa, indicate that fragmentation negatively impacts genetic diversity and structure (Neraas and Spruell 2001; Yamamoto et al. 2004; Jordan and Snell 2008; Lindsay et al. 2008; Sato and Harada 2008), and our study supports the same conclusion. Recent reports of change in population structure over the temporal scale have ranged from little change or no significant increase in subdivision (Johnson et al. 2004) to significant increase in differentiation (Martinez-Cruz et al. 2007) as observed in our study. Settlement of Texas and Oklahoma, ranching and railway development in the 1890s and early 1900s (Oklahoma 2005), may have caused the first fragmentation of the black-capped vireo habitat, with continuing massive-scale land conversion because of agriculture and urbanization, changing the natural landscape over much of central Texas (Schmidly and Sansom 2002). While losses of genetic diversity have been clearly associated with bottlenecks (Hauser et al. 2002; Paxinos et al. 2002), the evidence for reductions of genetic diversity within and increased levels of differentiation between populations isolated by anthropogenic fragmentation have been less clear-cut. Rapid loss of genetic diversity resulting from fragmentation similar to the extent found in our study has been reported in desert bighorn sheep (Epps et al. 2005) and in the Spanish imperial eagle (Martinez-Cruz et al. 2007).

### Coalescent simulations

Coalescent approaches have become useful tools for uncovering important demographic events in the history of populations (Mondol et al. 2009). In our case, coalescent simulations and ABC estimations suggest that a bottleneck occurred around 67 generations ago (from 2005). This equates to the decade of 1930–1940. The size of the bottleneck suggests that the population declined to approximately 10% of the prebottleneck value. A brief survey of Texas natural history failed to provide major anthropogenic habitat changes during the 1930s that may have contributed to a population crash, other than continuing habitat modification, loss and fragmentation. However, the timeframe coincides with the occurrence of the ‘1930s Dust Bowl’ (Schubert et al. 2004) with most of the decade characterized by extreme drought, dust storms and catastrophic vegetation failure, with the environmental repercussions presumably tapering out over the next decade. Dramatic climate change was pointed out by Darwin (1859) to be an important source of mortality and, hence, of variance in reproductive success. The following quote from *The Origin of Species* (1859) is particularly prescient and relevant to our study: ‘Climate plays an important part in determining the average numbers of a species, and periodical seasons of extreme cold or drought, I believe to be the most effective of all checks. I estimated that the winter of 1854–1855 destroyed four-fifths of the birds in my own grounds; and this is a tremendous destruction, when we remember that 10% is an extraordinarily severe mortality from epidemics with man. The action of climate seems at first sight to be quite independent of the struggle for existence; but in so far as climate chiefly acts in reducing food, it brings on the most severe struggle between the individuals, whether of the same or of distinct species, which subsist on the same kind of food’.

While pulses of freakish climate change can undoubtedly spike mortality, the genetic and evolutionary consequences of drawn-out climate change will need to be investigated – especially when such changes are also occurring concurrently with anthropogenic habitat changes. It has been argued that the severity of the 1930s Dust Bowl was exacerbated by human land use changes over the preceding 50 years (Worster 2004), which included suppressing natural fires, development of urban areas and conversion of forest into farmland. We postulate that this drastic climatic change event preceded by a period of disturbance and followed by continuing habitat loss and heavy cowbird parasitism may have led to prolonged periods of suppressed population sizes and, hence, declining genetic diversity. The estimated bottleneck had a credibility interval spanning a 50-year period between approximately 1920 and 1970, and this could be suggestive of the period over which the effects of the bottleneck persisted.

### Estimates of *N*_e_

The ML*N*_*e*_ approach assuming migration produced smaller estimates and wider confidence intervals compared to other estimators that assumed closed populations. It is important to note that the ML*N*_*e*_ joint estimator assumes that rates of migrants are from an infinitely large population, gene flow remains constant over the sampling interval, or there is no significant subdivision among source populations. We now know from analysis of structure that these assumptions are not satisfied. However, most natural populations do not persist with constant migration rates over time (Fraser et al. 2007). Nonetheless, it is likely that fluctuating migration rates and differentiating, noninfinite source populations may cause a stronger signal of drift, resulting in a lower estimate of *N*_e_. This offers an informative contrast with the approaches assuming closed populations. Bias has been a major concern with estimation of *N*_e_ based on temporal variance. However, these biases are expected to be less problematic with larger sample size, multiple sampling points, increasing number of variable loci and increasing the interval between samples. Palstra and Ruzzante (2008) report that small sample sizes are a major contributor to bias and low precision. Among our estimates involving historical samples, two of the three populations studied (Bexar and Oklahoma) had small sample sizes, and estimates involving these samples are likely to be biased. In the light of this, we have more confidence in the estimates of *N*_e_ from Kerr, for which population we have the best sample representation. ONeSAMP results also confirmed the trend that we observed with the ABC analysis, although actual estimates were higher for both the historical and contemporary samples.

Overall, our results from the various approaches discussed here, such as decreases in allelic diversity, heterozygosity within populations, increased differentiation among populations and evidence for a demographic bottleneck based on coalescent simulations, suggest that a population decline of considerable effect has occurred between the historical and contemporary period. Furthermore, these demographic changes coincide with documented environmental and habitat changes that have occurred over the last century. Based on these results, a strong case can be made for the need to improve genetic connectivity between remnant populations, if adverse genetic consequences or loss of adaptive potential are to be prevented. Further, the steep trajectory of genetic decline indicates that this species may have a diminished buffering capacity against continued onslaughts on their habitat.

Our work presented here provides a broadly applicable approach to address questions about population genetic change in cases where determining anthropogenic impacts may be valuable to future management. While use of archived genetic material is by no means novel, advances in molecular and computational methods make the use of limited archived tissue viable and especially valuable from a conservation perspective. We recently applied this approach successfully in revealing potentially critical details about the recent demography of another endangered species, the golden-cheeked warbler (Athrey et al. 2011). This work has spurred discussion on whether future management should consider managing genetic diversity.

## Implications and conclusion

Here, we were able to trace the genetic consequences of demographic change based on changes in allele frequencies over time, and by modeling alternative evolutionary scenarios. The ability to infer demographic history allows us to view genetic change in the context of habitat loss and fragmentation, and prognosticate on how this genetic change may impact the future persistence of populations.

Black-capped vireo populations are rebounding from their low census numbers following intensive cowbird control and active management of habitat, including prescribed burns, to suit their habitat requirements (Wilkins et al. 2006). As a testament to these management efforts, populations at Fort Hood and Kerr WMA (two places with cowbird trapping and prescribed burn programs) have both expanded dramatically – although it remains to be seen how this will reflect on future genetic analysis. Our data indicate that genetic decline and increasing fragmentation may still be ongoing, requiring continued management to stem the slide. Parasitism by cowbirds is expected to increase with increasing fragmentation and forest edges (Goguen and Mathews 2000; Patten et al. 2006; Benson et al. 2010), and habitat changes over the past century appear to have been important precursors for parasitism of Black-capped vireo broods. There is some evidence that fire suppression may encourage parasitism by cowbirds (Clotfelter and Yasukawa 1999). Suppression of natural fires is a relatively recent phenomenon – following inhabitation and ranching of central Texas from the early 1900s. Given the importance of fire for a) maintaining the successional structure of the habitat and b) being a potential disruptor of brood parasitism, prescribed burns may be a necessary management tool for maintaining and improving genetic connectivity among black-capped vireos, and hence maintaining existing genetic variability.

Although the northern range of the species has contracted, it is believed that the breeding range extends southward farther than previously thought (Farquhar and Gonzalez 2005). Our studies have not examined breeding populations in Mexico; hence, we urge caution in extrapolating our results to the entire species. Nevertheless, we were able to show that the differentiation observed by Barr et al. (2008) was not the historical norm for the species. Furthermore, we detected a loss of genetic diversity in the species, associated with a demographic bottleneck – neither of which was detected examining only contemporary samples (Barr et al. 2008). This result suggests that careful consideration is required when interpreting the time period to which single-sample genetic estimates apply. The responses of genetic parameters to demographic changes may be dependent on several factors unique to each species – including generation lengths, migration rates and fine-scale social structure. In some cases, single-sample estimates may not be sufficient to resolve demographic history, only revealing parameters that have had ample time to respond. At least for some analyses, a ‘false-negative period’ (Luikart et al. 1998; Cristescu et al. 2011) may obscure genetic signals of demographic change from being resolved from single samples. Quantifying genetic change with two or more temporal samples offers a resolution of both the trajectory and the time-scale on which these changes have occurred. These two attributes are likely to be best predictors of future genetic patterns and evolutionary implications thereof.

Other similar studies (Johnson et al. 2004; Martinez-Cruz et al. 2007) have shown that incorporation of temporal samples, when available, provides crucial insights important to conservation and are informative about evolutionary patterns in small populations. These studies utilized data on temporal genetic change to correlate with documented demographic declines. Martinez-Cruz et al. (2007) were able to detect an ancestral panmictic population and fragmented contemporary populations in the Spanish imperial eagle and suggest that restoration of the historical state should be incorporated into management plans. Johnson et al. (2004) found loss of genetic variation because of drift in Greater Prairie Chickens, resulting from habitat contraction. Along with these studies, our work emphasizes the importance of using temporally spaced data for evaluating how small populations fare over evolutionary time. This approach not only enables reliable assessments of genetic change over time, but also provides timely information that can be vital for species conservation.

Querying the past with ancient or archived DNA provides information that helps resolve details of the otherwise hazy evolutionary past of populations or species. Inferences from such analyses allow us to evaluate which events of the past may be most consequential for the evolutionary future of populations.

## Data Archiving

Data deposited in the Dryad repository: doi:10.5061/dryad.2r030k5p

## Supporting Information

**Table S1.** Comparison of changes in differentiation observed between historical and contemporary periods based on different estimators.

**Table S2.** Temporal estimates of the effective population size (*N*_e_) based on historical and contemporary samples, assuming open populations and a generation length (*T*) of 2 years.

**Appendix S1.** Details of laboratory methods used in processing archived tissue samples and methods of quality control.

**Appendix S2.** Details and results of coalescent simulations to examine loss of genetic diversity at constant population size.
