# Supplementary material for: Birds in space and time: genetic changes accompanying anthropogenic habitat fragmentation in the endangered black-capped vireo (Vireo atricapilla)
Source: Evol Appl. 2012 Jan 24;5(6):540–52. doi: 10.1111/j.1752-4571.2011.00233.x (PMC3461138; doi:10.1111/j.1752-4571.2011.00233.x)
Supplement: Supplementary file 1 [file eva0005-0540-SD1.doc]

**APPENDIX I**

**Extraction**:

All samples were randomized and blind labeled prior to processing. Owing to the very small quantity of tissue, we were only able to perform a single extraction step on all archived material. All DNA extraction from museum species was performed using a QiaAmp microextraction kit (Qiagen, Valencia, CA ), under a sterile hood in a laboratory where no contemporary samples had been processed.

**Amplification**:

Pilot amplifications of each sample were performed by Polymerase Chain Reaction (PCR) at each of 12 microsatellite markers developed specifically for this species . Generally, PCR conditions were similar to those reported in Barr et al Following this process, unreliable loci and samples were discarded and thermoprofile and chemical optimizations for each locus was established. Each PCR was set-up with a positive and negative control. Additionally, PCRs on historical samples were performed using a different set of pipettes than those used for contemporary samples.

**Genotyping**:

All genotyping was performed on an ABI 3130 Avant system, with Genescan software (Version 3.1, Applied Biosystems, Foster City, CA, USA). Allele calling was automated with the binning function and was checked manually.

***Reliability of genotyping protocol and error rates***

Of the 69 historical specimens examined, microsatellites from only 61 could be consistently amplified. Three of the 12 loci could not be amplified in any of the historical specimens. Hence all analyses reported are based on data from 9 microsatellite loci. Analysis of our (quadruple) replicate dataset with RELIOTYPE showed that 3 samples out of 61 (5%) did not meet the 95% reliability criteria (average reliability 88.3 ±4.1%), and up to 4 additional replications were recommended to meet the number of additional replicates required to observe each allele at least 2 times. After implementing suggested replications, our overall reliability across loci was established to be 98.4±0.9%. This translates into the possibility of fewer than 20 mistyped alleles in a total of 1098 alleles called. We felt that this potential error rate presented a very low risk of biasing our analysis solely due to genotyping errors. Analysis of our replicate dataset in GIMLET showed that the estimated error rate due to allele dropout was <2% across samples. The program MICROCHECKER found no evidence for null alleles in our consensus dataset and this data was used in further analysis.

Barr, Kelly R, Guha Dharmarajan, Olin E Rhodes Jr., Richard Lance, and Paul L Leberg. 2007. Novel microsatellite loci for the study of the Black-cappped Vireo (*Vireo atricapilla)*. *Molecular Ecology Notes* 7 (6):1067-1069.

Barr, Kelly R, Denise L Lindsay, N.R. Giridhar Athrey, Richard F Lance, Timothy J Hayden, Scott A Tweddale, and Paul L Leberg. 2008. Population structure in an endangered songbird: maintenance of genetic differentiation despite high vagility and significant population recovery *Molecular Ecology* 17 (16):3628-3639.
