# Supplementary material for: Birds in space and time: genetic changes accompanying anthropogenic habitat fragmentation in the endangered black-capped vireo (Vireo atricapilla)
Source: Evol Appl. 2012 Jan 24;5(6):540–52. doi: 10.1111/j.1752-4571.2011.00233.x (PMC3461138; doi:10.1111/j.1752-4571.2011.00233.x)
Supplement: Supplementary file 2 [file eva0005-0540-SD2.doc]

APPENDIX II

| **Estimate** | **Sites** | **Historic** | **Contemporary** | **T** | ***P*** |
| --- | --- | --- | --- | --- | --- |
|  | Bexar-Kerr | 0.0131 | 0.0461 | 2.54 | 0.017 |
| ***F_ST_*** | Kerr-Oklahoma | 0.0116 | 0.0348 | 3.33 | 0.005 |
|  | Bexar-Oklahoma | 0.0126 | 0.0497 | 3.15 | 0.006 |
|  | Bexar-Kerr | 0.0065 | 0.0254 | 2.59 | 0.017 |
| ***G_ST_*** | Kerr-Oklahoma | 0.0059 | 0.0184 | 2.30 | 0.025 |
|  | Bexar-Oklahoma | -0.0055 | 0.0126 | 4.63 | 0.001 |
|  | Bexar-Kerr | 0.1073 | 0.2897 | 1.90 | 0.030 |
| ***G'_ST_*** | Kerr-Oklahoma | 0.0164 | 0.2478 | 1.86 | 0.043 |
|  | Bexar-Oklahoma | -0.1696 | 0.1136 | 2.91 | 0.010 |
|  | Bexar-Kerr | 0.1046 | 0.2800 | 1.96 | 0.030 |
| ***D_EST_*** | Kerr-Oklahoma | 0.104 | 0.2412 | 1.91 | 0.040 |
|  | Bexar-Oklahoma | -0.1612 | 0.1024 | 2.79 | 0.010 |
|  |  |  |  |  |  |

Table S1: Comparison of changes in differentiation observed between historical and contemporary periods based on different estimators. Columns show different estimators, the combination of sites used in pairwise comparison of differentiation, Historic and corresponding Contemporary estimates of differentiation, T value reported from a paired t-test and the P value used to assess statistical significance at α=0.05.
