# Supplementary material for: Birds in space and time: genetic changes accompanying anthropogenic habitat fragmentation in the endangered black-capped vireo (Vireo atricapilla)
Source: Evol Appl. 2012 Jan 24;5(6):540–52. doi: 10.1111/j.1752-4571.2011.00233.x (PMC3461138; doi:10.1111/j.1752-4571.2011.00233.x)
Supplement: Supplementary file 3 [file eva0005-0540-SD3.doc]

**APPENDIX III**

Table S2: Temporal estimates of the effective population size (*N_e_*) based on historical and contemporary samples, assuming open populations and a generation length (*T*) of two years. Values shown are estimates of the likelihood estimates and corresponding 95% confidence intervals are shown.

| **Site** | | **Time** | | **No. Gen** | | **ML*N_e_*** | | **95% CI** | | ***m*** | | **95% CI** |
| --- | --- | --- | --- | --- | --- | --- | --- | --- | --- | --- | --- | --- |
| **Historical-Contemporary Comparisons** | | | | | | | |  | |  | |  |
| 1910-2005 | 47.5 | | 38 | | 18-100 | | 0.079 | | 0.021-0.241 | |  | |
| **Kerr** | | 1915-2005 | | 45 | | 105 | | 38-224 | | 0.046 | | 0.024-0.231 |
| **Oklahoma** | | 1910-2005 | | 47.5 | | 30 | | 14-85 | | 0.081 | | 0.031-0.245 |
| **Contemporary Sample Comparisons** | | | | | | | |  | |  | |  |
| **Kerr** | | 2005-2008 | | 1.5 | | 12 | | 11-15 | | 0.465 | | 0.327-0.614 |
