# Supplementary material for: Birds in space and time: genetic changes accompanying anthropogenic habitat fragmentation in the endangered black-capped vireo (Vireo atricapilla)
Source: Evol Appl. 2012 Jan 24;5(6):540–52. doi: 10.1111/j.1752-4571.2011.00233.x (PMC3461138; doi:10.1111/j.1752-4571.2011.00233.x)
Supplement: Supplementary file 4 [file eva0005-0540-SD4.doc]

**APPENDIX IV**

*Coalescent simulations to relate changes in heterozygosity to the effective size*

We used a simulation based approach to infer the probable population parameters that could have resulted in current population genetic estimates. Using the program BayesSSC (Bayesian Serial SimCoal) which is an implementation of the serial coalescent, we determined the population sizes at which the observed reductions (15%) in heterozygosity start becoming likely. Simulations were repeated 1000 times to generate heterozygosity values over the sampled history of the population. Each set of 1000 simulations were started assuming different *N_e_*, which varied from 50 – 25000 (total 14 different sizes). The range of *N_e_* was chosen arbitrarily to represent extremely small values to a maximum value that approaches the current *N_c_*. The starting *N_e_*’s are based on the haploid size*.* Coalescent phylogenies were generated based on 9 microsatellite loci at two mutation rates –high and low rates of 1.56 × 10^-3^ and 1.56 × 10^-4^ /generation respectively . Simulations were run assuming either *T*=1 or 2, resulting in either 90 or 45 generations between samples, reflecting the range in likely number of generations between our temporal samples collected in 1915 and 2005). However, as the analysis was not sensitive to variation in T, we only present results based on *T*=1.

*Results of Serial Coalescence Simulations*

We conducted coalescence simulations to test whether observed decline in genetic diversity (change in *H_EXP_* =15%) could be replicated under a model of constant population size (Fig S1). The results showed that such a decrease would only be probable at greater than 5% frequency when effective sizes were under 4000, under both mutation scenarios; however such a large decrease was never common under any of the population sizes modeled. These results were similar under both generation lengths used, so only those based on *T* =1 are shown.

Serial coalescent simulations can be very informative in confirming possible population histories that led to present conditions, especially when genetic data is available from multiple time points in history . Our simulations suggested that the probability of observing as great a change in heterozygosity (15%) was unlikely at effective sizes larger than 4000. However, even at simulations of much smaller *N_e_*, such a large change in *H_EXP_* was never common. Using the formula *H_T_*=*H_0_*[1-1/2*N_e_*]*^T^* where heterozygosity at time *T* (*H_T_*) can be calculated from initial heterozygosity (*H_0_*), given the population size *N_e_*, after *T* generations, a *N_e_* of 250 over 90 generations would result in a 15% decline in average heterozygosity that we observed in our data. Obviously if the period of population restriction was less than 90 generations, the *N_e_* would have been considerably smaller to result in a comparable decline in *H_EXP_*.

It is well understood that heterozygosity responds more slowly to bottlenecks compared to allelic richness . The large decreases in allelic richness and the estimates of variance *N_e_* indicate a severe bottleneck between historic and contemporary samples. So while it is possible that a constant *N_e_* as high as 4000 could produce a decline of 15% in heterozygosity, it is more likely that it was much smaller. Given that the whole breeding population in the US was estimated to be 190 breeding pairs in 1986, it is likely that the *N_e_* was this size or smaller for some period of time.

Figure S1: Results from coalescent serial simulations to test the hypothesis of constant population size. The simulations model change in heterozygosity based on different starting effective sizes at two different microsatellite mutation rates: (A) high with 0.00156 changes/generation and (B) low with 0.000156 changes/generation. The horizontal line represents the observed change in heterozygosity over a 90 year period.


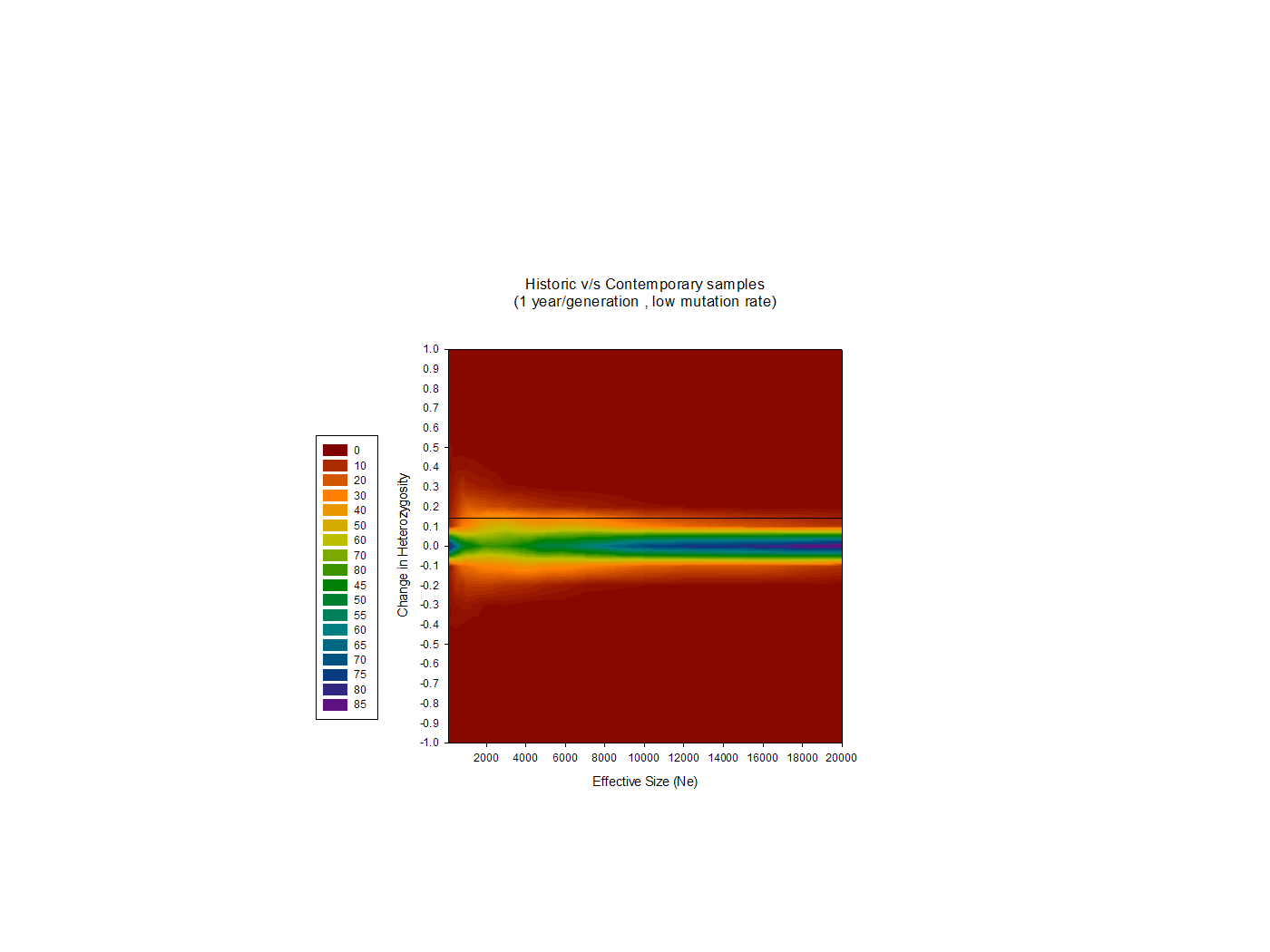

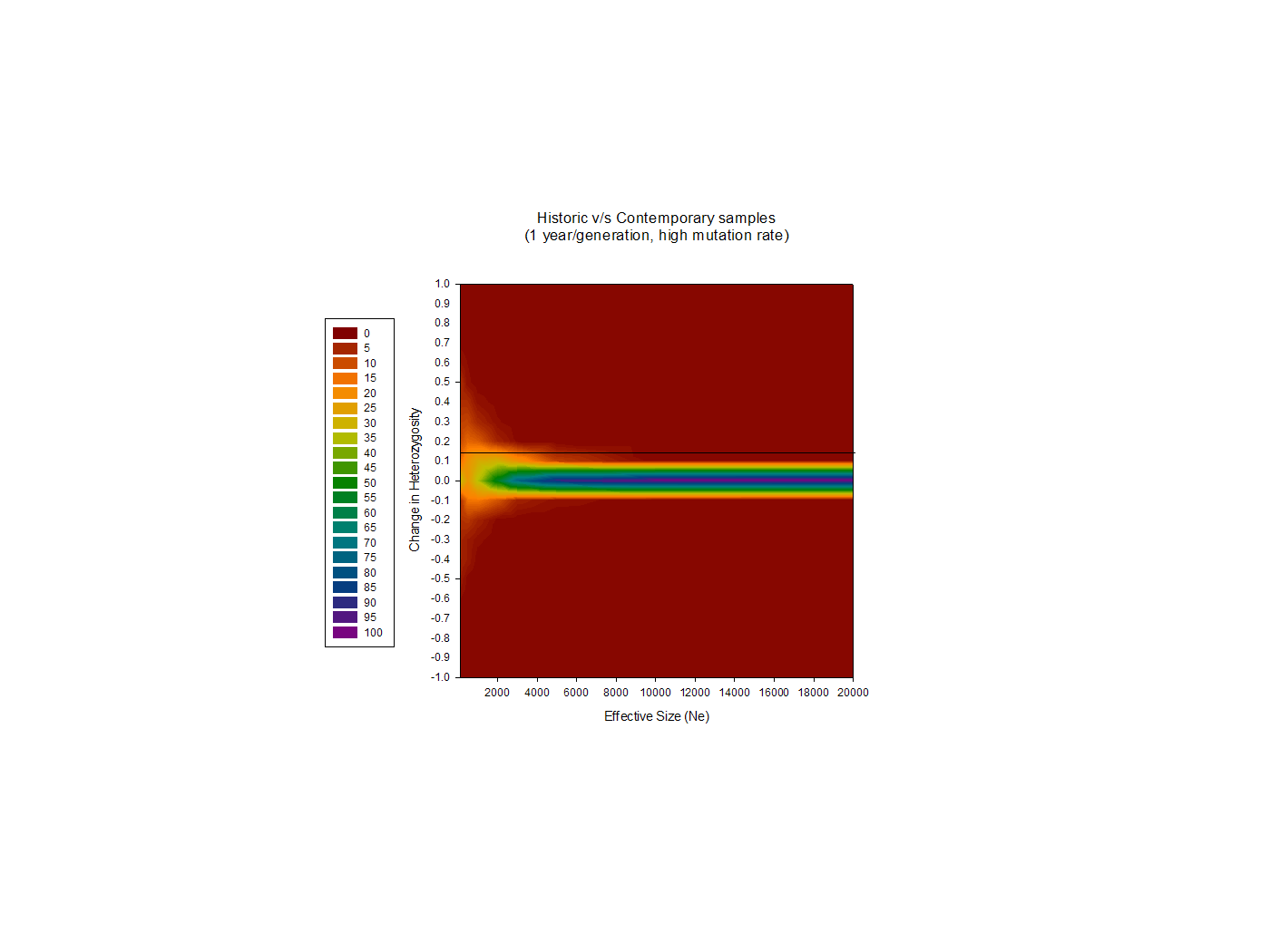


**Literature Cited**

Anderson, CNK, Uma Ramakrishnan, YL Chan, and EA Hadly. 2005. Serial SimCoal: A population genetic model for data from multiple populations and points in time. *Bioinformatics* 21:1733-1734.

Brohede, J, A P Moller, and H Ellegren. 2004. Individual variation in microsatellite mutation rate ini barn swallows. *Mutation Research* 545 (1):73-80.

Chan, Yvonne L., Christian N. K. Anderson, and Elizabeth A. Hadly. 2006. Bayesian Estimation of the Timing and Severity of a Population Bottleneck from Ancient DNA. *PLoS Genetics* 2 (4):e59-e59.

Kimura, Motoo, and James F Crow. 1963. The measurement of effective population number. *Evolution* 17 (3):279-288.

Laval, G, and L Excoffier. 2004. SIMCOAL 2.0: A program to simulate genomic diversity over large recombining regions in subdivided population with a complex history. *Bioinformatics* 21:2485-2487.

Nei, Masatoshi, Takeo Maruyama, and Ranajit Chakraborty. 1975. The bottleneck effect and genetic variability in populations. *Evolution* 29 (1):1-10.

Primmer, C, N Saino, A P Moller, and H Ellegren. 1998. Unraveling the processes of microsatellite evolution through analysis of germline mutations in barn swallows (*Hirundo rustica*). *Molecular Biology and Evolution* 15:1047-1054.

Spencer, Christine C, Joseph E Neigel, and Paul L Leberg. 2000. Experimental evaluation of the usefulness of microsatellite DNA for detecting demographic bottlenecks. *Molecular Ecology* 9 (10):1517-1528.

Wright, Sewall ed. 1968. *Variability within and among natural populations*. 4 vols. Vol. 4, *Evolution and the genetics of populations*

Chicago: The University of Chicago Press.
